# Inflammatory mediator modulation with specific or selective adsorbents

**DOI:** 10.1186/cc9673

**Published:** 2011-03-11

**Authors:** A Schildberger, T Buchacher, V Weber, D Falkenhagen

**Affiliations:** 1 Danube University Krems, Austria

## Introduction

Modulation of inflammatory mediators with specific or selective adsorbents may represent a promising supportive therapy for septic patients. The aims of this study were to compare the influence of specific or selective polymeric adsorbents on endothelial cell activation and to test various adsorbents for binding of high mobility group box 1 (HMGB1), a late mediator in sepsis.

## Methods

Human umbilical vein endothelial cells (HUVEC) were activated with a conditioned medium that was obtained by stimulation of monocytic THP-1 cells with 10 ng/ml lipopolysaccharide from *Pseudomonas aeruginosa *[1]. Mediator modulation was performed with either a specific adsorbent for TNFα, which is based on sepharose particles functionalized with anti-TNFα antibodies, or a selective albumin-coated polystyrene divinylbenzene copolymer (PS-DVB). Endothelial cell activation was monitored for up to 15 hours by measuring secretion of IL-6 and IL-8, as well as surface expression of the adhesion molecules ICAM-1 and E-selectin. In addition, PS-DVB beads and cellulose sulphate beads were screened for the binding of HMGB1.

## Results

Adsorption of inflammatory mediators from the conditioned medium either with the specific TNFα adsorbent or with the selective PS-DVB beads resulted in decreased endothelial cell activation, as shown by statistically significant reduction of IL-6 and IL-8 secretion from HUVEC, as well as statistically significant reduction of surface expression of the adhesion molecules ICAM-1 and E-selectin. In the screening experiments, both PS-DVB beads and cellulose sulphate exhibited strong adsorption of HMGB1. Studies to test the effect of HMGB1 removal on endothelial activation in the cell culture model are underway.

## Conclusions

Inflammatory mediator modulation with specific or selective adsorbents reduces endothelial cell activation and thus may support the development of new therapies for sepsis. Hydrophobic PS-DVB resins as well as cellulose sulphate exhibit strong adsorption of HMGB1, a late mediator of sepsis.

## References

[B1] Schildberger Innate Immun 2010 16 278 287 10.1177/1753425909341885 19710092

